# Motivations of patients with dentofacial deformities seeking orthognathic treatment: an application of Q methodology

**DOI:** 10.3389/froh.2026.1724098

**Published:** 2026-06-11

**Authors:** Keda Cao, Ziyi Yan, Shuzei Wang, Bin Zhang, Xiaoyi Hu, Xiaofeng Bai

**Affiliations:** 1 Key Laboratory of Shaanxi Province for Craniofacial Precision Medicine Research, College of Stomatology, Xi'an Jiaotong University, Xi'an, China; 2 Laboratory Center of Stomatology, College of Stomatology, Xi'an Jiaotong University, Xi'an, China; 3 Department of Head and Neck Tumor Surgery, College of Stomatology, Xi'an Jiaotong University, Xi'an, China; 4 Department of Oral and Maxillofacial Surgery, Hospital of Stomatology, China Medical University, Shenyang, China; 5 Department of Cranio-Maxillofacial Trauma and Plastic Surgery, College of Stomatology, Xi'an Jiaotong University, Xi'an, China; 6 Department of Oral and Maxillofacial Surgery, The Affiliated Hospital of Stomatology, Zhejiang University, Hangzhou, China

**Keywords:** dentofacial deformity, orthodontics, orthognathic surgery, Q methodology, surgical motivation

## Abstract

**Objectives:**

This study aims to systematically investigate the motivations behind orthognathic surgery in dentofacial deformity patients using Q methodology, thereby providing valuable insights for clinical decision-making in dentofacial deformity treatment. Additionally, the research seeks to evaluate the efficacy of Q methodology in subjective opinion surveys within this specific medical context.

**Methods:**

This exploratory, cross-sectional Q-methodology study (without pre-specified hypotheses) was conducted at the Department of Oral and Maxillofacial Surgery, Hospital of Stomatology, China Medical University, Shenyang, China, from December 2020 to December 30, 2021. Q methodology was employed to interview patients with dentofacial deformities to explore their motivations for surgery. The Q methodology comprises five main steps: 1. Q Statement Collection: Initially, the subject was defined, followed by the collection of motivation-related statements through literature reviews and interviews with patients with dentofacial deformities and orthognathic surgeons. 2. Q sample construction: Two professors of orthognathic surgery discussed and identified a Q sample, then tested it by a pre-experiment. 3. P sample selection: The quantity of the P sample depends on the Q sample and follows the principles of Q methodology. 4. Q-Sort: The P sample ranked the statements on an 11-point distribution grid ranging from “most agree” to “most disagree”. 5. Statistical analysis: Data processing and analysis were performed using the PQMethod2.35 program, and principal component analysis and factor analysis were carried out.

**Results:**

Through factor analysis, three distinct motivational viewpoints were identified, accounting for 67.5% of the total variance: Type 1, appearance-driven (27.5%); Type 2, occlusal function-dominant (17.5%); and Type 3, combined facial shape and occlusal function-driven (22.5%). Patients' explanations of their motivations for orthognathic surgery revealed that orthodontists' advice significantly influenced their decision to proceed with surgery.

**Conclusion:**

In this study, three distinct motivational profiles were identified among patients seeking orthognathic surgery: appearance-oriented, occlusal function-dominant, and a combined type. Awareness of these profiles may facilitate patient-centered communication and help align clinical discussions with patient expectations. Orthodontists' recommendations played an important role in patients' decision-making. These findings suggest that Q methodology represents a promising approach for investigating patient motivations in clinical settings.

## 1 Introduction

Dentofacial deformity is an abnormality in the morphology and position of the jaws (1). Patients with dentofacial deformities suffer from abnormalities in facial appearance and oral function, which significantly impact their quality of life (2). Orthognathic surgery is an important treatment method for dentofacial deformities (3), with the development and advancement of hemodynamics (4), surgery (5), and anesthesiology (6) in recent decades, the safety of orthognathic surgery has gradually improved and is now widely performed (7) in more hospitals. With the increase in the number of orthognathic surgeries, the motivation of patients has gradually attracted attention (8), and existing studies have demonstrated the correlation between surgical motivation and postoperative satisfaction in patients with dentofacial deformities (9).

As an elective procedure, orthognathic surgery is driven by various motivations (10). The degree of patient cooperation during treatment is directly linked to their motivation, influencing treatment outcomes (9, 11). In fact, researchers have been exploring the motivation of orthognathic surgery in dentofacial deformity patients (12), but the research on the treatment motivation of these patients is incomplete. At present, the investigation of surgical motivation is mainly conducted through questionnaires (8) or interviews (9), which usually have the following disadvantages: First, existing studies often require a large sample size to obtain relatively accurate results, which requires significant manpower and material resources (13). Second, in order to be “objective”, the way these survey methods are expressed, collected, and statistically analyzed is restricted, which may limit the participants’ responses (14). Moreover, this method of investigation isolates the subject's point of view, and participants respond to different views alone, limiting the variability of the results and thus masking the relative differences in different views (15). With the rapid development and changes in society, the existing questionnaires and scales often fail to keep pace with societal changes.

The Q methodology is a psychological research method that combines qualitative and quantitative research (16), which is more suitable for exploratory research on subjective opinions. People's views on a given topic may vary widely. However, representative views are limited, and those captured from a small, diverse sample are also present in larger populations (17). Therefore, the Q methodology requires obtaining as comprehensive a range of views as possible during the experimental design, so that even with a small sample size, general conclusions about behavioral motivation can be explored.

The main steps of the Q methodology include obtaining the Q set from the respondents' statements and data collection. The obtained Q sets are analyzed, and similar statements are grouped to form Q samples. The research subjects are selected as the *P* sample, and after the *P* sample is determined, the *P* sample ranks the Q statements. The obtained Q-sort data were analyzed, and the results were interpreted. The consensus opinions of the respondents can be obtained by having the research subjects rank the opinion statements related to the research topic, and the analysis of the Q-sort data can be carried out using specialized software such as PQMethod. The Q methodology has been widely used in psychology, communication, political science, journalism, and other fields (18).

As a subjective research method that combines qualitative and quantitative research, the Q methodology studies the relationships among individuals, focuses on sampling the research subjects, and emphasizes the subjectivity of the research subject (19). The Q methodology can reduce the influence of researcher subjectivity, allowing more comprehensive and accurate revelation of attitudes and opinions. Compared with traditional qualitative research, the Q methodology enables objective analysis through principal component analysis and factor analysis, avoiding the omission of viewpoints or misinterpretation (20). Accordingly, this exploratory, cross-sectional study employed Q methodology without pre-specified hypotheses to investigate the motivations for orthognathic surgery among patients with dentofacial deformity. The motivational patterns were subsequently refined to categorize patients into distinct profiles. This study is expected to offer valuable insights for physicians during surgical planning and doctor-patient communication.

## 2 Materials and methods

### 2.1 Sample collection

This study was approved by the Medical Ethics Committee of the Stomatological Hospital of China Medical University (Approval No. 2021037). Informed consent was obtained from all participants, and the consent forms were signed prior to the investigation. Based on the sample selection principle of the Q methodology, the number of included samples was determined. Patients who underwent orthognathic surgery and visited the Department of Oral and Maxillofacial Surgery at the Affiliated Hospital of Stomatology, China Medical University, between December 2020 and December 2021 were selected for inclusion in the study. Interviews were conducted after the patients signed the surgical informed consent forms.

#### 2.1.1 Inclusion criteria

1. Skeletal malocclusion and age over 18 years;
2. A clear willingness to undergo orthognathic surgery and currently undergoing treatment;
3. Voluntary participation in the questionnaire survey after fully understanding the purpose and process of the study;
4. Ability to comprehend and complete the questionnaires.

#### 2.1.2 Exclusion criteria

1. Cleft lip and palate, tumors, or secondary deformities resulting from maxillofacial trauma;
2. A history of genetic disorders affecting craniomaxillofacial development;
3. A history of orthognathic surgery;
4. A history of facial plastic surgery or injectable treatments;
5. Inability to understand the content of the scale or withdrawal from the survey during the completion process;
6. A history of mental illness or a family history of mental illness.

### 2.2 Research methodology

**Figure 1:**
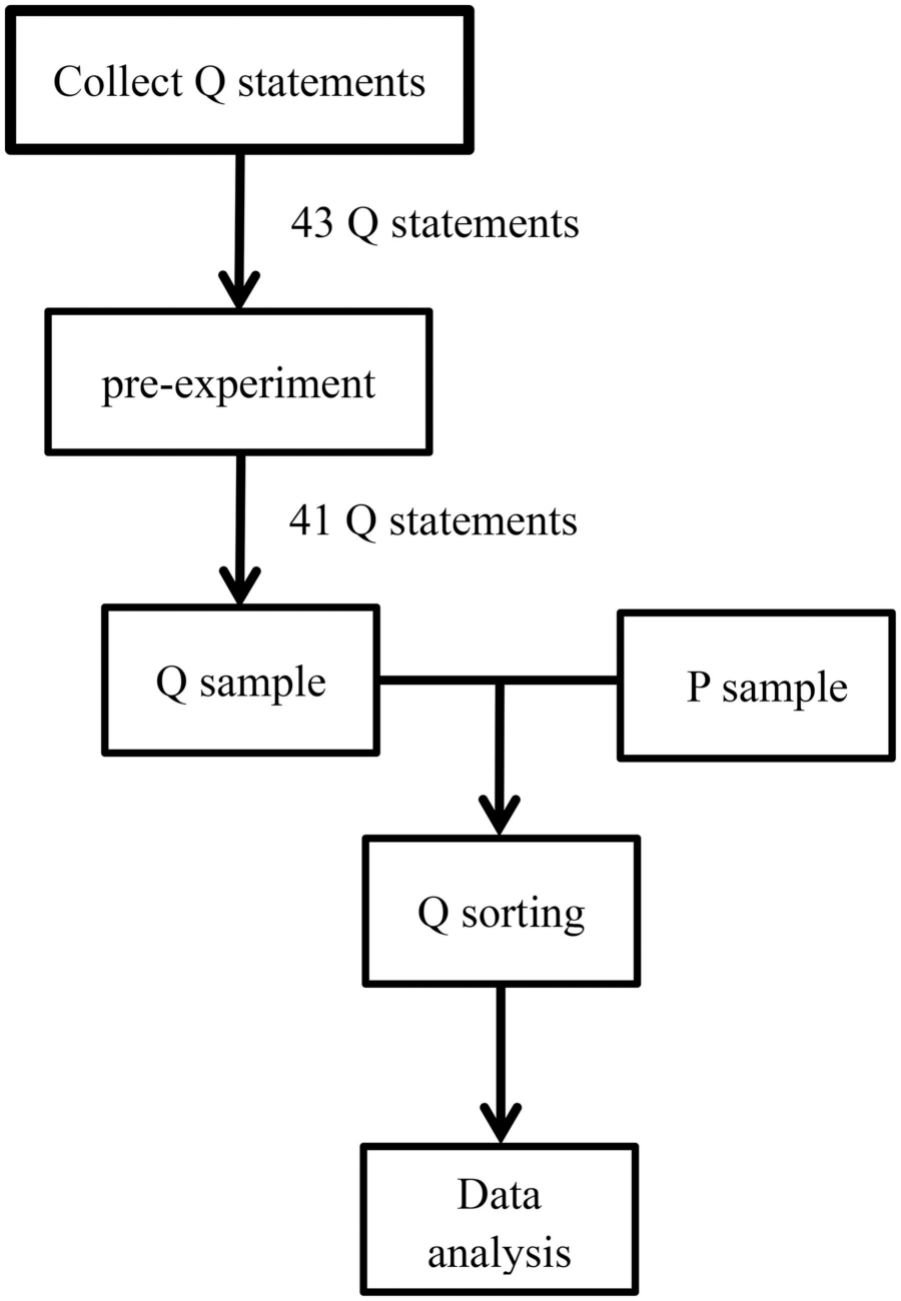
Flow chart of the Q methodology.

Figure 1 illustrates the Q-methodology workflow used in this study.

#### 2.2.1 Collect Q statements

The Q-sample was developed through a systematic, multi-stage iterative process to ensure its comprehensiveness, representativeness, and clarity, following established practices in Q-methodology (21).

An initial pool of statements (the concourse) was generated through three complementary sources:

- Systematic literature review: A systematic search was conducted in PubMed, CNKI, and Web of Science using the keywords “orthognathic surgery,” “dentofacial deformity,” “patient motivation,” and “treatment decision.” Studies reporting patient motivations for orthognathic surgery were retrieved, and verbatim statements describing motivational factors were extracted.
- Semi-structured patient interviews: Patients who met the inclusion criteria were recruited for individual interviews. Using the open-ended question “Why did you choose to undergo orthognathic surgery?” participants were encouraged to freely express their thoughts and concerns. Interviews continued until no new motivational themes emerged. All interviews were audio-recorded and transcribed verbatim, and relevant statements were extracted.
- Clinical expert consultations: Five senior orthognathic surgeons and orthodontists (each with >10 years of clinical experience) were consulted to capture clinical observations regarding common patient motivations that may not be fully reflected in the literature or patient interviews.

After preliminary deduplication and screening by the research team, a total of 52 distinct statements were retained to form the initial concourse.

#### 2.2.2 Development of Q samples

Step 1: Expert review and content validation

Two independent senior orthognathic surgery experts (chief physicians, each with >15 years of experience), who had not participated in the statement collection phase, were invited to evaluate the 52 statements. Each expert assessed the statements against four predefined criteria:

Relevance: The statement directly pertains to motivations for orthognathic surgery.

Clarity: The statement is unambiguous and easily understood by patients.

Uniqueness: The statement captures a distinct motivational aspect not redundant with others.

Balance: The overall set adequately covers the spectrum of potential motivations (functional, aesthetic, psychological, social).

Each expert independently rated the statements. After independent rating, the experts met to reach a consensus through discussion. This phase resulted in the deletion of six redundant statements, the consolidation of three groups of seven closely related statements into three new items, and the addition of two important motives identified by the experts as previously omitted (e.g., “fear that the deformity will worsen”). The statement set was thus adjusted from 52 to 43.

Step 2: Pilot testing and final refinement

To assess the feasibility and comprehensibility of the 43 statements, a pilot study was conducted with five patients who met the inclusion criteria. Participants completed the Q-sort and then underwent a cognitive interview. The pilot revealed that two statements were difficult to understand due to awkward phrasing (subsequently revised); one pair of statements was merged because of substantial semantic overlap; and all participants completed the sorting within 20 min. Based on this feedback, three statements were reworded for greater clarity, one pair of statements was merged, and the final Q sample was established, comprising 41 statements. All statements are phrased in the first person and in the affirmative (e.g., “I want to improve my facial profile”) to ensure ease of understanding and sorting. The complete final Q sample is presented in the Appendix.

#### 2.2.3 P sample selection

To obtain as much data as possible, 40 patients with dentofacial deformities who underwent orthognathic surgery in the Department of Oral and Maxillofacial Surgery, Hospital of Stomatology, China Medical University, were included in the study. The participants were selected to cover a wide range of ages, family backgrounds, economic conditions, educational backgrounds, and work statuses.

#### 2.2.4 Q sorting

**Figure 2:**
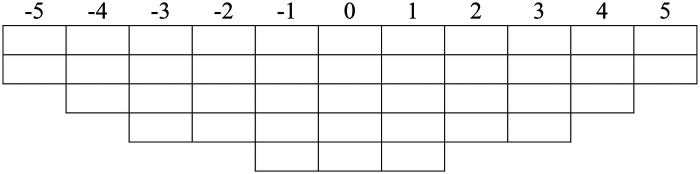
A Q-sort grid containing 41 statements.

Each participant assigned a ranking position to each statement through Q-sorting. Based on the theoretical framework, the 41 statements in the Q sample, and references to relevant literature, an 11-rank normal distribution table was initially selected (Figure 2). Each participant was asked to carefully read and understand all Q statements and then arrange them into a Q-sort table. The Q-sort form in this study consists of 11 ranking values, ranging from +5 (for statements with which participants most agree) to −5 (for statements with which they most disagree), with 0 indicating statements they are uncertain about. Participants were instructed to sort the statements into 11 columns. Additionally, each column could contain only one statement. Participants were permitted to rank the statements according to personal relevance and given unlimited time to place all cards in the Q-sort form. After completing the Q-sort, the researcher asked the participants (*P* sample) to explain their ranking decisions, particularly for the statements at the two extremes of the distribution table, to gain a detailed understanding of their perspectives.

#### 2.2.5 Data analysis

Data processing and analysis were performed using PQMethod 2.35 software. Principal component analysis (PCA) was conducted on the correlation matrix of the 40 Q-sorts. Factors with eigenvalues greater than 1.00 were initially considered for retention. Varimax rotation was applied to clarify the factor structure and maximize the variance explained by each factor (22). Factor loadings were considered significant at the *p* < 0.01 level, with the significance threshold calculated as 2.58 × (1/√N), where N equals the number of statements in the Q-sample (19). Factors retained for final interpretation were required to demonstrate both statistical adequacy (e.g., a minimum of four significant defining Q-sorts) and substantive interpretability (20, 22). The application of these criteria is detailed in the Results section.

## 3 Result

### 3.1 General statistical results

The 40 participants came from Liaoning, Jilin, Shaanxi, Heilongjiang, Inner Mongolia Autonomous Region, Sichuan and other provinces and cities, and the demographic information is shown in Table 1.

**Table 1:** Descriptive statistics of the study sample (*N* = 40).

Characteristic	Category	*N*	%
Gender	Male	14	35.0
	Female	26	65.0
Age, years	<20	8	20.0
	20–30	24	60.0
	>30	8	20.0
Level of education	Below undergraduate	20	50.0
	Undergraduate	15	35.0
	Master or above	6	15.0
Marital status	Single	18	45.0
	Relationship	14	35.0
	Married	8	20.0
Annual household income(RMB)	<50 thousand	6	15.0
	50 thousand∼100 thousand	10	25.0
	>100 thousand	24	60.0
Orthognathic surgery	Single-jaw	27	67.5
	Double-jaw	13	32.5

N, number; RMB, Renminbi (Chinese Yuan).

### 3.2 Statistical results of Q sorting

Principal component analysis of the 40 Q-sorts yielded ten factors with eigenvalues greater than 1.00, collectively explaining 78.47% of the total variance (Table 2).

**Table 2:** Eigenvalues and variance explained by factors.

Factor	Eigenvalues	Explained variance (%)	Cumulative explained variance (%)
1	12.8240	32.0600	32.0600
2	4.3929	10.9822	43.0422
3	2.7764	6.9410	49.9832
4	2.3148	5.7869	55.7701
5	2.1550	5.3876	61.1577
6	1.8937	4.7343	65.8920
7	1.5381	3.8454	69.7374
8	1.2297	3.0743	72.8117
9	1.1703	2.9256	75.7373
10	1.0915	2.7288	78.4661

To determine the number of factors for rotation, the scree plot was examined, revealing a clear inflection point after the fourth factor. A four-factor solution was therefore initially retained for Varimax rotation. These four factors had eigenvalues of 12.82, 4.39, 2.78, and 2.31, respectively, and together accounted for 55.77% of the total variance.

Following rotation, factor loadings were evaluated for statistical significance. Table 3 presents the rotated factor loadings for all 40 participants.

**Table 3:** Factor loadings (*N* = 40).

ID	F1	F2	F3	F4	ID	F1	F2	F3	F4
P1	0.30	0.70	0.18	0.13	P21	0.12	0.60	0.14	0.03
P2	0.28	0.50	0.65	−0.17	P22	0.38	0.40	0.45	−0.11
P3	0.30	0.22	0.76	−0.02	P23	0.41	0.69	0.21	−0.04
P4	−0.01	0.56	−0.17	0.42	P24	0.34	0.31	0.29	−0.48
P5	0.00	0.20	0.75	0.12	P25	0.45	0.49	0.06	−0.51
P6	0.17	0.04	0.71	0.42	P26	0.63	0.03	−0.19	−0.48
P7	0.23	0.56	0.43	0.22	P27	0.61	0.23	0.39	−0.18
P8	0.47	0.01	0.23	0.11	P28	0.74	−0.18	0.29	0.12
P9	0.54	0.26	0.33	0.07	P29	0.40	0.24	0.25	0.18
P10	0.27	0.71	0.22	0.20	P30	0.68	−0.33	0.21	0.14
P11	0.39	0.16	0.29	0.68	P31	0.61	0.19	0.44	0.00
P12	−0.10	0.76	0.06	0.12	P32	0.68	0.10	0.15	0.04
P13	0.09	0.75	0.04	0.19	P33	0.49	−0.05	−0.02	0.39
P14	−0.36	0.61	0.05	0.15	P34	0.82	0.24	0.18	0.05
P15	0.06	0.01	0.75	0.07	P35	0.21	−0.02	0.78	0.01
P16	0.20	0.15	0.56	0.03	P36	0.78	0.16	0.28	−0.04
P17	0.14	0.16	0.65	0.09	P37	0.17	0.26	0.27	0.66
P18	0.21	0.18	0.47	−0.15	P38	0.62	0.14	0.32	0.18
P19	0.39	0.33	0.36	−0.04	P39	0.33	−0.08	0.50	0.24
P20	0.04	0.68	0.16	−0.21	P40	0.31	0.40	0.31	0.40

To ensure that subsequent factor interpretation was based on statistically robust and replicable viewpoints, which enhanced the validity of the identified motivational profiles, we examined the rotated four-factor solution. This examination revealed that Factor 4 was defined by only three participants with significant loadings (P11, P24, and P37). As this fell below the recommended minimum of four to five defining sorts per factor for stable interpretation (20), Factor 4 was considered insufficiently robust and therefore excluded from further analysis. This decision was further supported by the principle that factors retained should demonstrate both statistical adequacy and substantive interpretability (22).

Regarding cross-loading participants, an individual Q-sort was considered to load significantly on a factor if its loading exceeded the significance threshold of ±0.402. A Q-sort was classified as confounded (cross-loading) if it achieved significant loadings on two or more factors. In such cases, the participant was excluded from factor interpretation to maintain the purity of each factor's viewpoint. Seven participants in this study demonstrated significant cross-loadings (P2, P4, P6, P7, P23, P25, P26) and were therefore excluded from the final factor solution. Three participants did not load significantly on any factor (P19, P29, P40) and were also excluded, consistent with standard Q-methodological practice which prioritizes the identification of shared viewpoints over the inclusion of all participants (21).

The remaining three factors were defined by 11, 7, and 9 participants respectively, together accounting for 67.5% of the explained variance (27.5%, 17.5%, and 22.5% respectively). Thus, 13 participants (32.5% of the total sample) were not retained for factor interpretation. Table 4 summarizes the key characteristics of the three motivational profiles. The complete factor arrays for all 41 statements, including significance levels for distinguishing statements, are provided in the Appendix (Table A1).

**Table 4:** Motivational Profile for Factors 1 to 3.

Characteristic	F1 Appearance-oriented	F2 Occlusal function-dominant	F3 Combined motivation
Sample size (N)	11	7	9
Explanation variance	27.5%	17.5%	22.5%
Eigenvalues	12.8240	4.3929	2.7764
Mean age (years)	24.3	27.4	23.6
Gender (M/F)	5/6	3/4	2/7
Primary motivation	Facial appearance and self-confidence	Masticatory function and health concerns	Both appearance and function
Key agreement statements	No chin is not beautiful (+5) I want to be more pretty (+5) I want to be more confident (+4) I want to look better in pictures (+4) I want to laugh better (+4)	It affected my eating (+5) Front teeth cannot bite well (+5) I want my teeth to bite better (+4) Afraid deformity will worsen (+4) Get surgery early while young (+4)	No chin is not beautiful (+5) It doesn't look good in mirror (+5) It affected my eating (+4) Afraid deformity will worsen (+4) I hope I feel better (+4) I want to be more pretty (+3)
Key disagreement statements	Relatives had surgery (-5) Friends suggest treatment (-4) Friends had surgery (-4) Slurred speech (-4) Look like idol (-3)	Relatives had surgery (-5) Low self-esteem (-5) Find a good partner (-4) Look like idol (-4) Reluctant to talk to people (-4)	Look like idol (-5) Solve before going abroad (-5) Media publicity (-4) Show gingiva when laugh (-4) Friends suggest treatment (-4)

F, factor; N, number; M/F, male/female.

#### 3.2.1 Factor 1: appearance-oriented patients

Factor 1 captured the viewpoint of 11 patients (27.5% of the variance), comprising 5 males and 6 females with a mean age of 24.3 years. This group primarily sought orthognathic surgery to enhance their facial appearance and self-confidence, with particular emphasis on dynamic aesthetics.

Their highest-rated statements focused on chin appearance (“No chin is not beautiful”, +5) and overall prettiness (“I want to be more pretty”, +5). Self-confidence was a central concern (“I want to be more confident”, +4), and they placed strong importance on how they appear in social contexts, including photography (“I want to look better when take pictures”, +4) and smiling (“I want to laugh better”, +4). Mirror reflection was also a significant motivator (“It doesn't look good when you look in the mirror”, +3).

Notably, this group consistently rejected external influences. They strongly disagreed that relatives' experiences (“Relatives have undergone orthognathic surgery”, −5) or friends' suggestions (“Friends suggest me come for treatment”, −4; “I have friends gone orthognathic surgery,” −4) played any role in their decision. They also dismissed functional concerns such as slurred speech (−4) and were not motivated by wanting to look like idols (−3).

#### 3.2.2 Factor 2: occlusal function-dominant patients

Factor 2 captured the perspective of 7 patients (17.5% of the variance), including 3 males and 4 females with a mean age of 27.4 years—the oldest among the three groups. These patients were primarily motivated by functional impairments and health concerns.

Their defining concerns centered on eating difficulties (“It affected my eating”,+5), compromised biting (“Front teeth cannot bite well”, +5), and the desire for better occlusion (“I want my teeth to bite better”, +4). Unlike Factor 1, this group expressed significant anxiety about disease progression (“Afraid that deformity will become more severe”, +4) and a sense of urgency about receiving treatment while young (“Get surgery done early while young”, +4).

This group was receptive to external professional input, with other doctors' recommendations playing a notable role (“Other doctors recommend me orthognathic surgery”, +3). In contrast, they strongly rejected psychosocial motivations: low self-esteem was rated as completely unimportant (−5), as was finding a good partner (−4). They also dismissed wanting to look like idols (−4) and were not reluctant to talk to people because of their teeth (−4).

#### 3.2.3 Factor 3: combined motivation patients

Factor 3 represented the viewpoint of 9 patients (22.5% of the variance), comprising 2 males and 7 females with a mean age of 23.6 years—the youngest group. These patients anticipated comprehensive life improvements from orthognathic surgery, expecting enhancements in both facial aesthetics and oral function.

Their highest-rated statements reflected a dual focus on appearance: mirror reflection (“It doesn't look good when you look in the mirror”, +5) and chin aesthetics (“No chin is not beautiful”, +5) were paramount, alongside functional concerns (“It affected my eating”, +4). Psychological well-being was prominently featured (“I hope I feel better”, +4), and they expressed significant concern about deformity progression (+4). Self-esteem issues were acknowledged (“Because of my teeth, my self-esteem is not very high”, +3; “A little low self-esteem”, +3).

Like Factor 1, this group strongly rejected trend-driven and external motivations. They dismissed wanting to look like idols (−5), solving problems before going abroad (−5), media publicity about orthognathic surgery (−4), and friends' suggestions for treatment (−4). Gingival display when laughing was also not a concern (−4).

## 4 Discussion

The primary motivations for orthognathic surgery among patients with dentofacial deformities include improving occlusal function, enhancing dental and facial aesthetics (23), and preventing future oral health issues. Using Q methodology and principal component/factor analysis, we categorized the motivations for orthognathic surgery into three types: 1. Appearance-oriented, 2. Occlusal function-dominant, and 3. A combination of facial aesthetics and occlusal function. The findings obtained using Q methodology are consistent with existing research (24) and are considered credible.

Factor 1 is named appearance-oriented and comprised the largest number of patients. This suggests that this motivational profile is the most prevalent. Additionally, the average age of these patients was close to the overall average age of orthognathic surgery patients. With the advancement of information technology, the vast majority of patients could access information related to orthognathic surgery through the Internet (25); most patients included in Factor 1 reported consulting online resources before visiting the clinic. However, these patients also noted that the existing online information is complex, which, while helpful, can also lead to confusion. As a result, patients still prefer to obtain information from medical professionals. Nevertheless, cognitive differences between medical professionals and patients persisted, indicating that doctor-patient shared decision-making (26) still has significant potential for improving doctor-patient communication.

According to Aditi Singh, some patients are unaware of their dentofacial deformities in daily life and only seek orthognathic surgery after being diagnosed with dentofacial deformities at a dental clinic (27). In China, public awareness of oral health care has significantly improved, and the popularization of oral science has achieved remarkable results. However, the dissemination of knowledge about oral diagnosis and treatment remains inadequate. Dental anxiety is one of the reasons patients refuse dental treatment, with descriptions of dental procedures by relatives and friends, as well as their own unpleasant dental experiences, being the primary causes of dental fear (28). The use of Q methodology to interview patients can help reduce their anxiety levels (29), thereby improving their compliance during the consultation process and ultimately enhancing satisfaction.

Factor 2 is named occlusal function-dominant, and the average age of patients in this factor was the highest among the three categories. Most of these patients were employed, and their income level was the highest among the three groups. Many patients had previously consulted about orthognathic surgery several years ago but decided against it due to concerns about surgical risks. However, they were now seeking treatment again due to the deterioration of occlusal function or temporomandibular joint disorders, leading them to reconsider their previous decision and accept orthognathic surgery. Although these patients tend to be more rational in their decision-making, this did not necessarily translate to higher postoperative satisfaction. The complexity of occlusal relationships and the temporomandibular joint, the most intricate joint in the human body, made disease progression challenging to predict. Studies have shown that postoperative satisfaction among occlusal function-dominant patients is lower than that of appearance-oriented patients (30). Therefore, it was crucial to communicate thoroughly with such patients before treatment, explaining the complexity of occlusal relationships and managing their expectations. Additionally, some patients in this category also suffer from sleep apnea syndrome. The risks associated with sleep apnea syndrome have gradually gained increasing attention.

Some patients were concerned about the length of hospital stay. According to reports from the United Kingdom and Iceland (31), bilateral sagittal split ramus osteotomy (BSSRO) can be performed in a day ward (32), significantly reducing the duration of hospitalization. Currently, orthognathic surgery in China is primarily conducted in public hospitals. Due to considerations of surgical safety and various medical policy requirements, patients typically needed to stay in the hospital for 3 to 5 days. Cost is another common concern for patients in this category, which can influence their decision to undergo surgery. While orthognathic surgery is covered by medical insurance in many European countries, most orthognathic surgeries in China are not eligible for insurance reimbursement. The cost of orthognathic surgery (33) is relatively high, and the financial burden is largely borne by the patients themselves.

Factor 3 is named facial shape and occlusal function combination. Patients in this category were the youngest among the three groups, with the highest proportion of female patients, the lowest average education level, the lowest annual family income, and the highest proportion of single individuals. These patients often exhibited signs of depression during treatment and investigation, but they were more willing to cooperate with the Q methodology investigation, which aligns with reports suggesting that the Q methodology can improve patient compliance (34). The improvement of self-confidence and quality of life is a delicate and complex process, making it appropriate and necessary to provide proper psychological counseling for such patients. Most of these patients first consulted an orthodontist, whose recommendation significantly influences their decision to pursue orthognathic surgery. Orthodontists are often the starting point for dentofacial deformity treatment (35), and it is essential for orthodontists and orthognathic surgeons to strengthen communication and collaboration.

With the development and advancement of artificial intelligence (36) and deep learning technology (37), we could explore the application of artificial intelligence (38) to communicate with dentofacial deformity patients in the future. Some literature suggests that the advice of family and friends plays a significant role in motivating patients with dentofacial deformities, particularly from acquaintances who have undergone orthognathic surgery (39), Such advice may directly influence the surgical decisions of these patients. However, in this study, the majority of patients were primarily motivated by internal factors. It has also been reported in the literature that specialized orthognathic surgeons play a crucial role in patients’ decisions to undergo orthognathic surgery (40).

Most existing studies on orthognathic surgery are based on qualitative research involving interviews. For instance, Torgersbråten et al. interviewed 60 patients with Type II high-angle cases and identified oral function as their primary motivation (30). Similarly, Mi et al. explored the influence of socio-cultural factors and physiological dissatisfaction on orthognathic decision-making (41). As a research method that combines qualitative and quantitative approaches, the Q methodology utilized principal component analysis and factor analysis to effectively quantify patients' perspectives, facilitating further statistical analysis. Tang et al. (14) employed the Q methodology to investigate the corrective motivations of patients with cleft lip and palate, as well as their parents, while Bryant et al. used the Q methodology to explore the core motivations behind repeated self-harm, identifying distinct functional profiles of the behavior (34). Furthermore, the Q methodology was a reliable research tool, with its reliability reaching up to 0.8 (18), Therefore, the Q methodology might be further developed into an evaluation tool to assess patients' motivations for orthognathic surgery. This tool can be directly applied to individual patients to evaluate their treatment motivations, enhance doctor-patient communication, formulate targeted treatment plans, and ultimately improve patient satisfaction and compliance.

This study has several limitations, which are presented as methodological limitations (related to study design and execution) and interpretive limitations (related to generalizability and clinical application).

### 4.1 Methodological limitations

First, all participants were recruited from a single center in Northeast China. Although the sample varied in age, sex, and educational level, the single-center design limits the generalizability of the findings to other regions or clinical contexts within China. Second, the Q-sort was administered on the day before surgery. While this timing ensured that participants had given informed consent and remained in treatment, preoperative anxiety can influence subjective responses (42). The present study did not measure anxiety levels at the time of sorting; thus, the motivational profiles may partly reflect transient emotional states rather than stable, long-term motivations. Future studies should administer the Q-sort at an earlier, less emotionally charged time point or include a standardized anxiety scale. Third, a certain rate of exclusion is inherent to Q-methodology. Of the 40 participants, 13 (32.5%) were not retained for factor interpretation (Factor 4 was defined by only three participants; seven participants showed cross-loadings; three had no significant loading on any factor). This rate is acceptable given the clinical heterogeneity of the sample, and each retained factor exceeded the recommended minimum of four to five defining sorts for stable interpretation (20, 22).

### 4.2 Interpretive limitations

First, motivations for orthognathic surgery are culturally sensitive. Prior studies have shown that aesthetic expectations differ across cultures: Asian patients tend to prefer a softer, smoother facial contour (43), whereas Western patients are more attuned to lateral profile and functional improvement (44). The motivational profiles identified in this study were derived from a Chinese patient sample and may not be directly generalizable to Western populations or other cultural settings without further validation. Second, as an exploratory, cross-sectional study, we did not establish direct associations between motivational types and clinical outcomes (e.g., postoperative satisfaction, treatment adherence, or long-term quality of life). Therefore, these motivational profiles should serve as a reference framework to facilitate patient-centered communication rather than as predictors of surgical outcomes. Clinicians should integrate these profiles with the patient's cultural background, individual circumstances, and clinical findings.

Future research should address these limitations. Multicenter, cross-cultural Q-method studies are needed to validate the generalizability of the motivational classification. Longitudinal studies should be conducted to establish associations between motivational types and postoperative outcomes. Qualitative interviews may also help deepen the understanding of how culture shapes patients' perceptions of aesthetics, function, and health.

## 5 Conclusion

Most patients in this study reported multifaceted treatment motivations, encompassing improvements in facial contour, occlusal function, and self-confidence, with one factor often emerging as dominant. Based on Q methodology and subsequent factor analysis, patients in the present sample were classified into three motivational profiles: appearance-oriented, occlusal function-dominant, and combined. Awareness of these profiles may facilitate patient-centered communication and enable orthognathic surgeons to better align clinical discussions with patient expectations, which may in turn contribute to improved treatment compliance and satisfaction. In addition, orthodontists' recommendations appeared to play an important role in the decision-making process among this patient population. These findings are broadly consistent with existing literature and suggest that Q methodology represents a promising approach for investigating patient motivations in clinical settings.

## Data Availability

The original contributions presented in the study are included in the article/Supplementary Material, further inquiries can be directed to the corresponding author/s.

## Appendix

**Table A1:** Complete factor arrays for all statements

Statement	Factor 1	Factor 2	Factor 3
1.I hope it helps in interpersonal interactions	0	1	2
2.I want to be more recognized in studies or work	−1	0	0
3.Slurred speech	−4	3	1
4.I want to find a good partner	0**	−4**	−2
5.Ugly teeth affects job hunting	−1	−3*	1
6.It affected my eating	−3**	5	4
7.Ugly teeth makes me very reluctant to talk to people	0	−4*	0
8.It doesn't look good when you look in the mirror	3*	2*	5*
9.I don't like the way my face looks right now	3	1	2
10.No chin is not beautiful	5	3	5
11.I hope I feel better	2	2	4
12.Orthognathic treatment can make me happier	1	3**	1
13.Because of my teeth, my self-esteem is not very high	2	1	3
14.I want to look better when take pictures	4	−1**	3
15.Relatives have undergone orthognathic surgery	−5	−5**	−3
16.A little low self—esteem	1	−5**	3
17.Afraid that deformity will become more severe	1**	4**	4**
18.Think my teeth get protrusion	0	2	1
19.Biting tongue when speak	−1*	−3	0
20.My parents wanted me to have orthognathic surgery	−2	1	0
21.I have friends gone orthognathic surgery	−4	−2	−1
22.I want to laugh better	4**	1	1
23.Friends suggest me come for treatment	−4	0**	−4
24.Other doctors recommens me orthognathic surgery	0	3**	−3**
25.I want to be more confident	4	0*	2**
26.I want to be more pretty	5	−1**	3
27.Orthognathic surgery can get more attention	−3	−1*	−3
28.Get surgery done early while young	2	4	−2**
29.I want to look like my favorite idol	−3	−4	−5
30.Front teeth cannot bite well.	−1**	5*	0**
31.Snoring while sleeping	−2	−2*	−3
32.Ridiculed by others because fo appearance	−1	−2	−1
33.Solve dental problems at home before go abroad	−3	−3	−5*
34.Show gingiva when laugh	2**	−3	−4
35.I want to achieve better in my work	−2	−1	−1
36.The media publicity about orthognathic surgery	−2	−2	−4
37.I want my teeth to bite better	3	4**	2
38.Don't show enough teeth when laugh	3	0	−1
39.I want to make more friends	−1	−1	−2
40.Orthognathic surgery help others become beautiful	1**	0	−2
41.Discomfort in the temporomandibular joint	1**	2	−1

Notes: * **p* ≤ 0.01; **p* ≤ 0.05.
