# Correction: Motivations of patients with dentofacial deformities seeking orthognathic treatment: an application of Q methodology

**DOI:** 10.3389/froh.2026.1907737

**Published:** 2026-06-23

**Authors:** Keda Cao, Ziyi Yan, Shuzei Wang, Bin Zhang, Xiaoyi Hu, Xiaofeng Bai

**Affiliations:** 1 Key Laboratory of Shaanxi Province for Craniofacial Precision Medicine Research, College of Stomatology, Xi'an Jiaotong University, Xi'an, China; 2 Laboratory Center of Stomatology, College of Stomatology, Xi'an Jiaotong University, Xi'an, China; 3 Department of Head and Neck Tumor Surgery, College of Stomatology, Xi'an Jiaotong University, Xi'an, China; 4 Department of Oral and Maxillofacial Surgery, Hospital of Stomatology, China Medical University, Shenyang, China; 5 Department of Cranio-Maxillofacial Trauma and Plastic Surgery, College of Stomatology, Xi'an Jiaotong University, Xi'an, China; 6 Department of Oral and Maxillofacial Surgery, the Affiliated Hospital of Stomatology, Zhejiang University, Hangzhou, China

**Keywords:** dentofacial deformity, orthodontics, orthognathic surgery, Q methodology, surgical motivation

In the published article, there was an error in the **Funding** statement. The original statement declared that no financial support was received:

“The author(s) declared that financial support was not received for this work and/or its publication.”

A correction has been made to the **Funding** section. The correct Funding statement is:

“The author(s) declared that financial support was received for this work and/or its publication. This work was supported by grants from the Key Research and Development Program of Shaanxi (2021SF-047), the National Key Research and Development Program of China (2019YFB1311604), and the Science and Technology Plan Project of Xi‘an, Shaanxi, China (25YXYJYB00089).”

The original version of this article has been updated.

